# Defining the molecular blueprint that drives CD8^+^ T cell differentiation in response to infection

**DOI:** 10.3389/fimmu.2012.00371

**Published:** 2012-12-19

**Authors:** Brendan E. Russ, Alice E. Denton, Lauren Hatton, Hayley Croom, Matthew R. Olson, Stephen J. Turner

**Affiliations:** ^1^Department of Microbiology and Immunology, University of MelbourneParkville, VIC, Australia; ^2^Department of Medicine, Cambridge Research Institute, Cambridge UniversityCambridge, UK

**Keywords:** cytotoxic T cells, memory T cells, transcription factors, epigenetics, histone modifications

## Abstract

A cardinal feature of adaptive, cytotoxic T lymphocyte (CTL)-mediated immunity is the ability of naïve CTLs to undergo a program of differentiation and proliferation upon activation resulting in the acquisition of lineage-specific T cell functions and eventual establishment of immunological memory. In this review, we examine the molecular factors that shape both the acquisition and maintenance of lineage-specific effector function in virus-specific CTL during both the effector and memory phases of immunity.

## INTRODUCTION

A cardinal feature of adaptive, cytotoxic T lymphocyte (CTL)-mediated immunity is the ability of naïve CTLs to undergo a program of differentiation and proliferation upon activation resulting in the acquisition of lineage-specific T cell functions and eventual establishment of immunological memory (Kaech et al., 2002a; van Stipdonk et al., 2003). CTLs contribute to the control and eventual elimination of a myriad of pathogen (intracellular bacteria and viruses) and tumor challenges via the coordinated interplay of varied effector mechanisms that include; (1) the production of pro-inflammatory cytokines such as interferon (IFN)-γ and tumor necrosis factor (TNF)-α (La Gruta et al., 2004); and (2) the expression of cytolytic effector molecules including perforin (Pfp; Kagi et al., 1994) and the granule enzymes (granzymes, Gzm) A, B, and K (Jenkins et al., 2007; Peixoto et al., 2007; Moffat et al., 2009). Once infection is cleared, the expanded effector T cell population contracts with establishment of a pool of long-lived, pathogen-specific memory T cells (Marshall et al., 2001; Kaech et al., 2002b; La Gruta et al., 2004). Although quiescent, memory CTLs demonstrate rapid effector function without the need for further differentiation (Lalvani et al., 1997; Oehen and Brduscha-Riem, 1998; Cho et al., 1999; Veiga-Fernandes et al., 2000). The combination of a high frequency and rapid effector function enables memory CTLs to respond more rapidly upon secondary infection, enabling earlier control and clearance of infection. Importantly, our understanding of the factors that not only shape the cell fate decisions to be a memory versus effector T cells are unclear, but the molecular mechanisms that enable stable maintenance of rapid effector function in the long-term are also not well understood. This review will examine new advances in our understanding of the molecular mechanisms that control effector and memory CD8^+^ T cell differentiation.

## INITIATION OF T CELL RESPONSES

Initiation of naïve CTL activation requires recognition of pMHC complexes on a specialized subset of antigen-presenting cells, termed dendritic cells (DCs). DCs exist as two general populations – tissue-resident and lymph node-resident DCs (Heath and Carbone, 2001). Importantly, in the context of peripheral infections such as respiratory influenza A virus infection and herpes simplex virus infection of the skin, both tissue-resident and lymph node-resident DCs appear to play roles in the induction of T cell immunity (Allan et al., 2003; Belz et al., 2004; Heath and Carbone, 2009). A primary role for tissue-resident DCs is continual surveillance of their environment for the presence of invading pathogens. Upon infection, tissue-resident DCs are activated via pathogen pattern receptors, such as Toll-like receptors or intracellular sensors such as RIG-I, MDA-5, and members of the inflammasome complex, resulting in activation and trafficking of these DCs from the tissues to the draining lymph node. The activation of DCs also results in the concomitant up-regulation of co-stimulatory molecules, such as CD80/CD86. Thus, these migratory DCs not only carry antigen from the infected tissue to the draining lymph node, but are now capable of providing the necessary secondary signals to promote activation of naïve and memory antigen-specific T cells (Heath and Carbone, 2001; Belz et al., 2007). A third signal, provided via signaling induced by pro-inflammatory cytokines such as type I IFNs and interleukin (IL)-12, is also required for full priming of mature effector T cell responses (Curtsinger et al., 2003a,b). Thus the integration of multiple signals received via the T cell receptor, co-stimulation and inflammatory cytokine receptors is required to drive differentiation of naïve T cells to effector and memory cells.

## THE EFFECTOR PHASE: ACQUISITION OF CTL EFFECTOR FUNCTION

As little as 2 h of *in vitro* peptide stimulation is sufficient to initiate an autonomous program of T cell proliferation and differentiation (Kaech and Ahmed, 2001; van Stipdonk et al., 2003). These initial observations were supported by *in vivo* studies demonstrating that early termination of antigen-presentation did not overly impact effector and memory CTL differentiation after infection (Wong and Pamer, 2001, 2003; Prlic et al., 2006). Thus, it would appear that naïve CD8^+^ T cells are pre-programed for differentiation prior to any antigen exposure. This concept is aligned with the recent data demonstrating that dynamic changes in genomic and transcriptional programing occurring during T cell development are key for establishing a genetic blueprint that likely underpins the fate of naïve T cells after activation (Zhang et al., 2012).

Progressive differentiation is a key factor that shapes both the phenotypic and functional heterogeneity of pathogen-specific CTL responses (Marzo et al., 2005; Badovinac et al., 2007). The acquisition of IFN-γ (Lawrence and Braciale, 2004), Pfp (Jenkins et al., 2008), and granzyme expression (Oehen and Brduscha-Riem, 1998; Jenkins et al., 2008; Moffat et al., 2009) is clearly linked to ongoing lymphocyte proliferation (Badovinac et al., 2007; Jenkins et al., 2008). In addition, functional profiling of effector and memory CTL induced after primary influenza A virus infection of C57BL/6J mice demonstrated that profiles of intracellular cytokine expression (both mRNA and induced protein) followed a strict hierarchy and most likely reflected sequential acquisition of multiple effector functions due to progressive differentiation following activation (La Gruta et al., 2004).

In terms of cytokine production, recent observations suggest that polyfunctional potential (TNF-α^+^IFN-γ^+^) is acquired within three to four divisions with acquisition of IFN-γ production (Denton et al., 2011). However, extended cycling leads to the loss of TNF-α production for a substantial set of activated CTLs leading to a progressive diminution in polyfunctional capacity. This is supported by the observation that activation of TCR transgenic T cells with “low affinity” ligands leads to an inability to sustain extended proliferation with these less differentiation CTL exhibiting co-expression of IFN-γ and TNF-α (Zehn et al., 2009). This contrasts with acquisition of cytolytic gene expression (Pfp and the granzymes) where continued cell division leads to a broader spectrum of effector gene expression (Jenkins et al., 2007, 2008; Peixoto et al., 2007). Recent data also suggests that there is a hierarchy of expression with granzyme B acquired early after activation with extended proliferation required for both GzmA and GzmK expression (Jenkins et al., 2008; Moffat et al., 2009; Zehn et al., 2009). Differences in the temporal expression of regulatory factors required for Gzm and cytokine gene loci likely explains these differences.

## THE MEMORY PHASE

Memory T cells can be broadly divided into “central” and “effector” memory subsets, with the two differing in both phenotypic and functional characteristics that reflect the different roles they play in response to secondary infection (Sallusto et al., 1999, 2004). Effector memory T cells (or T_EM_) typically express tissue-specific homing markers such as CCR5, CXCR3, and integrins and while they can be found in the circulation, significant numbers are found in the non-lymphoid tissues (Masopust et al., 2001). Moreover, T_EM_ are associated with decreased proliferative capacity and immediate effector function, such as cytotoxicity in the case of CTL (Masopust et al., 2001). While T_EM_ are capable of entering non-lymphoid tissues from the circulation in the steady state (Wakim et al., 2008; Kohlmeier et al., 2011), recent reports have identified tissue-resident T_EM_ that persist in the long-term at the original site of infection (Gebhardt et al., 2009, 2011; Mackay et al., 2012).

Central memory T cells (T_CM_) typically express the lymph node homing markers, CD62L (L-selectin) and CCR7, and exhibit greater proliferative capacity when compared to T_EM_ (Masopust et al., 2001). The fact that T_CM_ localize to lymph nodes in greater numbers and are capable of proliferation in response to secondary infection ensures greater numbers of effector CTL are generated earlier. Thus, T_CM_ provide a more rapid response and provide a second wave of effector CTL capable of clearing any remaining active infection that T_EM_ have failed to control (Wherry et al., 2003).

Just when after infection memory T cells are generated is the basis of some conjecture. However, there is strong evidence that T cell memory can be established very early after infection, especially when inflammation is limiting. For example, the prophylactic use of antibiotics prior to *Listeria monocytogenes* infection, or vaccination with peptide pulsed DCs, demonstrated that functional memory T cells can be generated as soon as 4–6 days after priming (Badovinac et al., 2005). This is supported by a study where it was shown that T cells isolated from IAV infected mice as early as 3–4 days after infection could form memory when adoptively transferred into a second host (Kedzierska et al., 2006, 2007).

Recent studies have suggested that effector or memory T cell fate can be predicted during the primary effector phase based on the cell surface expression of both the killer cell lectin-like receptor G1 (KLRG1) and IL-7 receptor subunit-α (IL-7Rα; Kaech et al., 2003; Joshi et al., 2007). Activated T cells that express high levels of KLRG1 and low levels of IL-7Rα (KLRG1^hi^IL-7Rα^lo^) are largely destined to be terminally differentiated effector cells and are termed short-lived effector cells (SLECs). In contrast, the small population of activated T cells that are KLRG1^lo^IL-7Rα^hi^ demonstrate memory potential and are termed memory precursor effector cells (MPECs). While the models of effector versus memory T cell fate are still an area of debate (reviewed by Kaech and Cui, 2012), recent advances have started to provide insights into the molecular factors that shape the outcomes of T cell activation.

## MOLECULAR FACTORS THAT SHAPE ACQUISITION OF EFFECTOR FUNCTION

**FIGURE 1:**
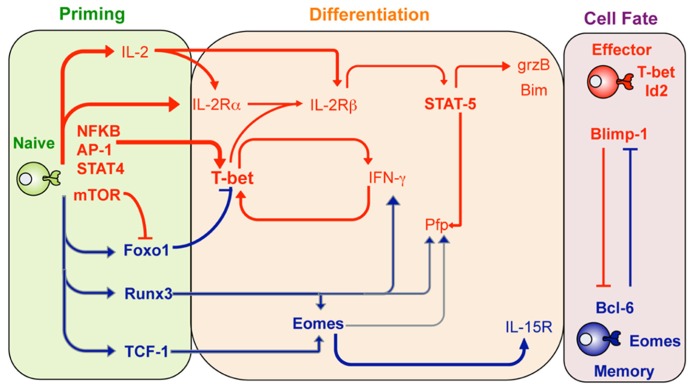
**Transcription factor regulation of effector and memory T cell differentiation**. Shown are transcription factors that are expressed and act to promote either effector CTL (red lines) or memory CTL (blue lines) differentiation at the different stages after infection including the priming phase (green), differentiation stage (orange) or once the cell fate has been determined (purple shading). Adapted from Pipkin and Rao, Cell, Snapshots Archive, Effector and memory T cells.

Specific transcription factors determine T cell effector and memory function and fate (Intlekofer et al., 2005; Joshi et al., 2007; Cruz-Guilloty et al., 2009; Kallies et al., 2009; Pipkin et al., 2010; Zhang et al., 2010; **Figure 1**). In the case of CTL effector function three transcription factors appear to play “pioneering” roles in determining effector T cell differentiation. Two T-box transcription factors, Tbx21 (T-bet) and Eomesodermin (Eomes; Intlekofer et al., 2005) play essential roles in effector CTL differentiation. While T-bet is normally associated with CD4^+^ T_H_1 lineage commitment, in part, by promoting expression of IFN-γ (Szabo et al., 2000), it is also rapidly up-regulated in activated CTL, contributing to rapid acquisition of IFN-γ production and helping promote GzmB expression (Cruz-Guilloty et al., 2009). Eomes, a homolog of T-bet, was originally implicated in the regulation of CD8^+^ T cell GzmB expression (Pearce et al., 2003), however recent studies suggest that Eomes is expressed later during CTL differentiation and contributes more to acquisition of Pfp expression and maintenance of the capacity to express IFN-γ (Cruz-Guilloty et al., 2009). The fact that Eomes over-expression does not rescue diminished GzmB expression in T-bet-deficient CTL suggests that the contribution of Eomes in determining specific T cell function is highly dependent on the timing and extent of expression (Cruz-Guilloty et al., 2009).

It is becoming clear that various transcription factors work cooperatively to reinforce the commitment of naïve T cells to become fully differentiated effector T cells. For example, the runt-related transcription factor 3 (Runx3), expressed by naïve CTLs, promotes expression of a variety of signature CTL effector molecules such as IFN-γ, GzmB, and Pfp (Cruz-Guilloty et al., 2009). Runx3 appears to have both a direct affect via binding to promoter and regulatory regions within the *Ifn*-γ, *gzmB*, and *pfp* gene loci. Moreover, Runx3 also promotes Eomes transcription further promoting CTL differentiation and acquisition of effector function (Cruz-Guilloty et al., 2009). Given that Runx3 is constitutively expressed in naïve T cells, just what regulates Runx3 activity is not clear. However, upon T cell activation Runx3 can bind to IL-2 responsive regulatory elements within the *pfp* gene locus (Cruz-Guilloty et al., 2009). Thus it is likely that IL-2 signals received upon CTL activation are required for Runx3 activity on a subset of effector gene loci.

Interleukin-2 is a key cytokine required for inducing proliferation and survival of activated T cells (Miyazaki et al., 1995). Importantly, high levels of IL-2 signaling at the time of CTL activation also contribute to signature effector gene expression, including expression of GzmB and Pfp (Janas et al., 2005; Pipkin et al., 2010). IL-2-dependent regulation of effector CTL differentiation is primarily via IL-2Rβ signaling and subsequent activation of STAT5. This results in binding of activated STAT5 to the GzmB promoter, thus helping promote gene transcription. In contrast, up-regulation of Pfp expression first requires STAT5 to bind to regulatory elements within the Eomes promoter with subsequent Eomes expression able to promote Pfp expression (Cruz-Guilloty et al., 2009). This IL-2-STAT5 pathway also likely explains the IL-2 dependency of Runx3 promotion of effector gene expression (Cruz-Guilloty et al., 2009).

Another key transcription factor in CTL effector differentiation is the B lymphocyte-induce maturation protein-1 (Blimp-1) encoded by *Prdm1*. A role for Blimp-1 in lymphocyte differentiation was first observed in activated B cells where it is required for the terminal differentiation and subsequent maintenance of long-lived antibody-secreting cells (Shapiro-Shelef et al., 2003). Recent studies have demonstrated that Blimp-1-deficient T cells are unable to fully differentiate into effector CTL in response to virus infection. Rather, Blimp-1-deficient CTL preferentially differentiate into effector CTL that have “memory like” characteristics such as high levels of IL-7α and Bcl6 expression (see below) and low levels of typical effector markers such as KLRG1 and GzmB (Kallies et al., 2009; Rutishauser et al., 2009). Thus, Blimp-1 appears to ensure that activated lymphocytes, including CTL, become terminally differentiated effectors. Importantly, the same high IL-2 activating conditions that contribute to CTL effector gene expression, also contribute to up-regulation of Blimp-1 and repression of the transcription factor Bcl6 and IL-7Rα expression (Pipkin et al., 2010). This transcriptional profile is a hallmark of terminal CTL differentiation (Kaech et al., 2002b) and hence IL-2 not only contributes to acquisition of effector function but also to effector CTL differentiation.

So what signals dictate the decision for activated CTL to differentiate toward either an effector or memory T cell fate? A major driver appears to the response of activated CTL to pro-inflammatory mediators produced upon infection. For example, T-bet up-regulation in activated CTLs is clearly induced via TCR ligation and inflammatory mediators such as IL-12 and IFN-γ (Mullen et al., 2001; Szabo et al., 2002; Sullivan et al., 2003; Takemoto et al., 2006), and results in differentiation of effector T cells. Moreover, IL-12 signaling serves to both simultaneously promote and suppresses T-bet and Eomes expression, respectively (Takemoto et al., 2006). Thus, the degree of inflammatory stimulation serves to establish higher levels of T-bet and tips the balance toward effector CTL differentiation (Joshi et al., 2007).

## REGULATING THE T-bet/Eomes NEXUS AND MEMORY T CELL FATE

Current evidences suggest that the programing of T cell memory occurs early during the priming phase (Feau et al., 2011). Thus, what precisely are the factors that translate signals received during priming into this memory capacity? While initially considered a key driver of signature CTL effector gene expression, and hence effector differentiation, it has recently emerged that Eomes may play a more prominent role in memory T cell formation and persistence (Intlekofer et al., 2005; Banerjee et al., 2010). This in part likely due to Eomes-dependent up-regulation of the IL-2β receptor (CD122), enabling responsiveness to IL-15, a cytokine needed for maintenance of memory T cells (Intlekofer et al., 2005).

As described earlier, induction of high levels of T-bet expression in response to pro-inflammatory cytokines serves to promote effector T cell differentiation. Thus, it is interesting that T-bet deficiency within virus-specific CTL results in fewer T_CM_ (Intlekofer et al., 2007). Thus, T-bet appears to not only promote effector T cell differentiation, but to actively suppress memory T cell formation. Just how T-bet does this is not clear but is tempting to speculate that it may act as a transcriptional repressor inhibiting expression of gene loci required for memory T cell programing. This will be a key area of research in the future.

The importance of regulating the T-bet/Eomes nexus in determining effector versus memory T cell fate is highlighted by the fact that a number of extrinsic signaling pathways serve to regulate the balance of T-bet and Eomes levels in activated T cells. Expression of the transcription factor Foxo1 actively represses effector differentiation by blocking T-bet expression, while at the same time promoting Eomes expression and maintenance of memory T cell generation (Rao et al., 2012). Activation of the mammalian target of rapamycin (mTOR) kinase inactivates Foxo1 function thereby releasing T-bet from Foxo1 inhibition and thus, promoting effector CTL differentiation (Rao et al., 2012). Therefore, the use of rapamycin, or some other inhibitor of mTOR activity may be a useful intervention that promotes memory T cell generation.

Another member of the fork head family of transcription factors, Foxo3a, has also been implicated in the regulation of effector versus memory T cell fate (Riou et al., 2007; van Grevenynghe et al., 2008). Comparison of transcriptional signatures between polyclonal human CD4^+^ T_CM_ and T_EM_ demonstrated differential expression of genes regulated by Foxo3a (Riou et al., 2007), including Bim. Phosphorylation results in the exclusion of Foxo3a from the nucleus and subsequent transcriptional inactivation (Brunet et al., 1999), thus it was of interest that T_CM_ had higher levels of phosphorylated Foxo3a compared to T_EM_. Importantly, it was determined that both TCR and signals via the common γ-chain cytokine receptor induce Foxo3a phosphorylation and subsequent protection from Bim-mediated apoptosis. Hence, this provides a molecular mechanism for how homeostatic signals regulate memory T cell persistence (Riou et al., 2007). Moreover, inhibition of Foxo3a expression has been shown to prolong human immunodeficiency virus-specific memory T cell survival, indicating that Foxo3a is a potential target for therapeutic intervention that could promote memory T cell establishment (Riou et al., 2007; van Grevenynghe et al., 2008).

In another example of extrinsic signals promoting memory T cell differentiation, activation of the Wnt-β-catenin signaling pathway has been shown to promote expression of the transcription factor T cell factor-1 (TCF-1), with subsequent up-regulation of Eomes (Zhou et al., 2010). As observed in Eomes-deficient mice (Banerjee et al., 2010), TCF-1-deficient mice fail maintain a CD62L^hi^ T_CM_ population after challenge, supporting the notion that programing of memory CTL requires expression of TCF-1 that in-turn promotes Eomes expression (Zhou et al., 2010).

## THE ROLE OF OTHER TRANSCRIPTIONAL REGULATORS IN MEMORY T CELL DIFFERENTIATION

Given that inflammation is a key driver of effector T cell differentiation, a question that arises is how does memory arise in the face of a robust infection? Recent evidence suggests that environmental cues may serve to limit the impact of inflammatory-driven effector T cell differentiation allowing for memory T cell formation early after infection. Both IL-10 and IL-21 have been reported to promote memory T cell differentiation (Yi et al., 2010). Further, it has been recently demonstrated that after LCMV infection, signaling via STAT3, a transcription factor necessary for transmitting IL-10R and IL-21R signals, is necessary for memory CTL formation (Cui et al., 2011). Significantly, STAT3-dependent signals promote the expression of the transcription factor BCL6, known to be up-regulated within memory CTL while at the same time, repressing Blimp-1 expression. Moreover, STAT3-deficient CTL had lower levels of suppressor of cytokine signaling-3 (SOCS-3) expression and were more responsive to IL-12-dependent differentiation (Cui et al., 2011). Thus, signaling via immune regulatory cytokines, such as IL-10 and/or IL-21, can promote memory T cell differentiation by both up-regulating key memory T cell fate governing genes, while at the same time actively limiting the impact of inflammatory signals and subsequent effector CTL differentiation.

Finally, the inhibitor of DNA binding (Id)-2, and Id3 proteins have opposing roles in determining effector versus memory CTL generation. While pathogen-specific CTL within Id2-deficient mice could respond and differentiate into effectors, they exhibited a diminished response magnitude after infection and delayed pathogen clearance (Cannarile et al., 2006; Yang et al., 2011). Importantly, there was a failure to establish a T_EM_ population upon clearance of infection. These data suggest that Id2 up-regulation is required for sustained effector differentiation and establishment of the T_EM_ repertoire. Moreover, STAT5 and STAT4 binding sites have been identified within the Id2 promoter suggesting that the inflammatory signals known to drive effector differentiation, such as IL-2 and IL-12, can act on Id2 to promote this fate (Yang et al., 2011).

In contrast to Id2, Id3 is down-regulated upon CTL activation but is then re-expressed by memory T cells (Yang et al., 2011). Interestingly, while effector differentiation is normal in Id3-deficient mice, there is a failure to maintain a long-lived memory T cell population upon clearance of infection (Ji et al., 2011). Thus, Id3 expression is a key checkpoint that contributes to establishment of a robust memory T cell population. In fact, up-regulation of Blimp-1 has been shown to inhibit Id3 expression, representing a key switch in effector versus memory T cell fate determination (Ji et al., 2011; Yang et al., 2011).

In summary, a complex transcriptional network interprets and integrates the various extrinsic signals received by an activated T cell soon after infection. While there is significant understanding about the precise role these transcriptional networks have in T cell differentiation, little is known about regulation of the genomic template they bind and how changes in the biochemical and structural composition of the genome regulates this activity. The next section of this review will examine the role of epigenetics, or modifications of chromatin, in regulating effector and memory T cell differentiation.

## EPIGENETIC REGULATION: THE ROLE OF HISTONE MODIFICATIONS

**FIGURE 2:**
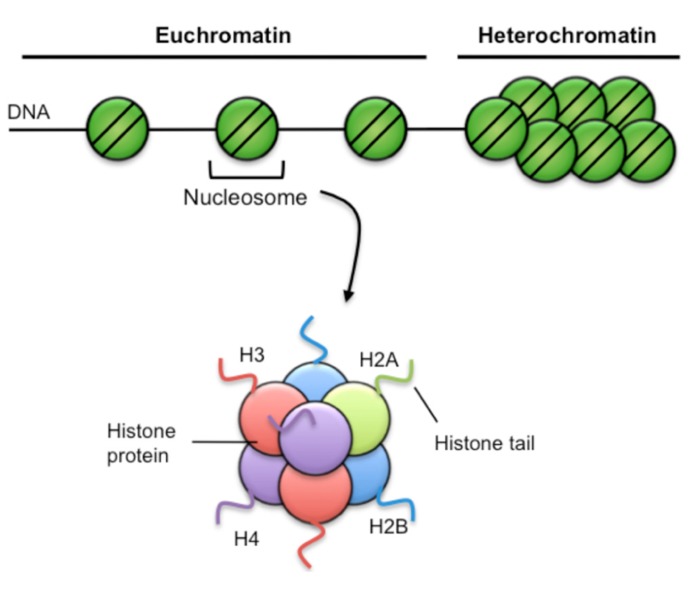
**Chromatin structure**. Chromatin has two broad structures. The first is euchromatin, characterized by sparse nucleosome density and is generally associated with active gene transcriptional activity. Heterochromatin is characterized by high nucleosome density, is very compacted and is generally associated with repression of gene transcription. Nucleosomes consist of 147 bp of DNA wound 1.65 turns around a complex of histone proteins, comprising two each of the H2A, H2B, H3, and H4 histone variants. Each histone has a soluble amino terminal tail that can be covalently modified by specific epigenetic marks such as acetylation, methylation, and phosphorylation.

Within eukaryotic cells, genomic DNA is wrapped around a complex of histone proteins that organize to form a structure termed chromatin (**Figure 2**). The basic unit of chromatin is the nucleosome, where DNA is wrapped around an octameric histone complex, typically containing two each of the core histones H2A, H2B, H3, and H4. Importantly, the composition of chromatin structure and biochemical modifications of histone proteins cannot only regulate short-term gene expression patterns within a cell, but can be propagated as a proliferates ensuring stable inheritance of a cellular phenotype, a process termed epigenetics.

Histone modifications in particular are thought to modulate gene expression by either changing chromatin structure and/or by providing a platform that promotes binding of transcriptional regulators (Kouzarides, 2007). Histone proteins can be modified by a vast array of covalent modifications, particularly on the solvent-exposed N-terminal tail with the combination of histone PTMs and their genomic location a predictor of transcriptional activity (**Table 1**; Zhang and Reinberg, 2001; Wang et al., 2008). For example, acetylation of the histone H3 at lysine 9 (H3K9Ac) that is associated with gene promoters is a positive correlate of transcriptional activation (Wang et al., 2008). Broadly speaking, histone acetylation is thought to promote a more open chromatin structure by masking the overall positive charge of histones (Aoyagi et al., 2002). This relaxes chromatin structure, and potentiates transcription factor binding and the recruitment of the core transcription machinery. Conversely, a lack of histone acetylation restricts chromatin accessibility and can render the DNA inaccessible to the transcriptional machinery (Ura et al., 1997).

**Table 1:** Histone modifications and their association with gene expression^a^.

Histone	Site	Modification	Activity^b^
H2A	S1	Phosphorylation	-
	K5	Acetylation	+
	K119	Ubiquitylation	+/-
H2B	K5	Acetylation	+
	K12	Acetylation	+
	K15	Acetylation	+
	K20	Acetylation	+
	K120	Ubiquitylation	+/-
H3	R2	Methylation	-
	K4	Methylation	+
		Acetylation	+
	K9	Methylation	-
		Acetylation	+
	S10	Phosphorylation	+
	K14	Acetylation	+
	R17	Methylation	+
	K18	Acetylation	+
	K23	Acetylation	+
	K27	Methylation	-
		Acetylation	+
	K36	Methylation	+/-
	K79	Methylation	+
H4	R3	Methylation	+
	K5	Acetylation	+
	K8	Acetylation	+
	K12	Acetylation	+
	K16	Acetylation	+
	K20	Methylation	-

a Adapted from Yi et al . (2010).

b Associated with activation (+) or repression (-) of genes.

Methylation of histone proteins is somewhat more complicated. For example, tri-methylation of histone 3 at lysine 4 (H3K4me3) is associated with almost all actively transcribed genes, and has a strong correlation with histone acetylation and recruitment of RNA polymerase II, indicative of a transcriptionally permissive gene (Bernstein et al., 2002; Santos-Rosa et al., 2002; Heintzman et al., 2007; Muse et al., 2007). Conversely, H3K4 dimethylation can be associated with either active or repressed genes (Bernstein et al., 2005), suggesting dual roles for this mark. In contrast to transcriptional activation, deposition of tri-methylation of H3K27 (H3K27me3) within promoter regions is strongly linked to transcriptional repression (Wang et al., 2008). Another repressive mark, H3K9me3, is implicated in heterochromatin formation through its interaction with heterochromatin protein 1 (HP1; Jacobs and Khorasanizadeh, 2002).

It is becoming increasingly evident that it is the combination and degree of enrichment of histone modifications that is key for fine-tuning gene transcription or silencing (Barski et al., 2007; Wang et al., 2008). The combination of different active and repressive histone modifications at specific loci defines the transcriptional state of individual genes (Strahl and Allis, 2000), thus defining cell fate. This is most evident when considering patterns of H3K4me3 and H3K27me3 within the same promoter regions. Genome-wide mapping of these two modifications in embryonic stem cells (ESCs) has demonstrated that regions important for maintaining ESC pluripotency are enriched for both H3K4me3 and H3K27me3, termed “bivalent” loci (Bernstein et al., 2006; Cui et al., 2009; Hawkins et al., 2010). Importantly, upon differentiation, the vast majority of bivalent loci within stem cells resolve to H3K27me3 ensuring that inappropriate gene expression within specific cell lineages does not occur (Bernstein et al., 2006). These data suggest that bivalency is a switch mechanism by which genes can be rapidly activated or repressed depending on the differentiation pathway initiated. 

## APPROACHES FOR THE ANALYSIS OF HISTONE MODIFICATIONS WITHIN THE GENOME

A standard approach for examining histone modification within the genome is chromatin immunoprecipitation. Initially, DNA–protein complexes within nuclei are cross-linked by formaldehyde fixation, followed by fragmentation of the DNA by enzymatic digestion or mechanical disruption (i.e., sonication). The DNA–protein complexes are then immunoprecipitated using antibodies specific for either DNA binding proteins (such as transcription factors) or specific histone covalent modifications. The purified complexes are then treated to reverse the cross-links, and the DNA isolated and used as a real-time PCR template to interrogate specific genomic regions of interest. The focused nature of this approach means that only small genomic regions are probed in any one reaction (typically 100–300 bp) with extensive analysis of a particular gene locus requiring a laborious and systematic approach.

The advent of next-generation sequencing technology has revolutionized the study of epigenetic modifications by enabling genome-wide profiling of chromatin modifications, an approach termed ChIP-seq (Kharchenko et al., 2008; Park, 2009). ChIP-seq involves “deep-sequencing” the immunoprecipitated DNA with the subsequent short sequences (or reads) being mapped back onto a reference genome. This approach has yielded an unprecedented level of resolution identifying not just the genomic location of specific modifications, but the specific patterns of enrichment, as well as their association with particular genomic features such as promoter and enhancers. In combining such data with large-scale transcriptional profiling (i.e., by microarray), our understanding of how epigenetic modifications underpin key cellular processes is undergoing a renaissance.

## EPIGENETIC REGULATION OF CD8^+^ T CELL EFFECTOR FUNCTION: ACQUISITION AND MAINTENANCE

A defining characteristic of T cell immunity is the acquisition of lineage-specific effector function that is readily maintained into memory. There is a large body of work has determined that specific epigenetic mechanisms underpin CD4^+^ effector T cell lineage commitment from a naïve state into different effector subsets (reviewed by Ansel et al., 2003; Kanno et al., 2012). Similarly, there is a growing body of work that has examined epigenetic regulation of CD8^+^ effector T cell differentiation.

Granzyme B expression by activated CD8^+^, but not CD4^+^ effector cells, generally reflects differences in the lineage-specific functions observed for the CD4^+^ and CD8^+^ T cell subsets. Recently we demonstrated that differences in GzmB expression by *in vitro* activated CD4^+^ and CD8^+^ T cells correlates with difference in epigenetic modifications within the *gzmB *locus (Juelich et al., 2009). While, CD8^+^ T cell expression of GzmB was coupled to a significant increase in chromatin accessibility, H3K9ac and H3K4me3 deposition, and docking of RNA polymerase II at the *gzmB* promoter region, few of these changes occurred within activated CD4^+^ T cells. Strikingly, this study suggests that differential programing of CD4^+^ and CD8^+^ T cell subsets during T cell development dictates the what lineage-specific effector function will be acquired upon activation. In the case of mature, naïve CD8^+^ T cells, it is most likely the combination of transcription factors including Eomes and Runx3 that direct acquisition of GzmB expression within activated CD8^+^, but not CD4^+^ T cells. It still remains to be determined whether these lineage-specific transcription factors play a role in directing the observed epigenetic changes during differentiation, or drive effector gene expression after chromatin remodeling has occurred.

In terms of acquisition and maintenance of CTL-specific functions recent analysis has demonstrated that dynamic changes in specific histone modifications can underpin observed phenotypic and functional changes during CD8^+^ T cell differentiation. It is under appreciated that naïve T cells can exhibit rapid effector function upon TCR ligation whereby they rapidly produce TNF-α, but not IFN-γ prior to initiation of division (Brehm et al., 2005; Priyadharshini et al., 2010; Denton et al., 2011). Co-expression of TNF-α^+^ and IFN-γ^+^ is observed within three to four divisions, but extended proliferation leads to the loss of TNF-α production for a substantial proportion of activated CD8^+^ T cells leading to a progressive diminution in polyfunctional capacity (Denton et al., 2011). Importantly, the capacity of naïve, effector and memory T cells to make IFN-γ and TNF-α is directly linked to the presence of defined epigenetic signatures within the proximal promoters (Denton et al., 2011). For example, the *tnfA* proximal promoter of naïve OT-I cells has an overall permissive epigenetic landscape with increased chromatin accessibility, enrichment for H3K4me3/H3K9ac and lack of H3K27me3. This is compared to the generally repressive epigenetic signature for the *ifnG* promoter with a closed chromatin structure, lack of H3K4me3/H3K9ac and enrichment of H3K27me3 (Northrop et al., 2006; Denton et al., 2011; Zediak et al., 2011).

The *ifn*-γ promoter region undergoes significant remodeling in effector CD8^+^ T cells, becoming more accessible and acquiring a permissive epigenetic signature (Northrop et al., 2006; Denton et al., 2011; Zediak et al., 2011). The fact that such events are required for the acquisition of effector gene expression and take time to eventuate probably explains the link between continued cell division and the acquisition of lineage-specific functional capacity within recently activated T cells (Lawrence and Braciale, 2004; Jenkins et al., 2008; Denton et al., 2011). While extended effector T cell differentiation leads to permissive epigenetic marks being deposited within the *ifn*-γ promoter to allow IFN-γ expression (Northrop et al., 2006; Denton et al., 2011; Zediak et al., 2011), there was progression to a more repressive *tnfA* epigenetic signature (decreased chromatin accessibility and increased H3K27me3 deposition; Denton et al., 2011). Importantly, the fact that both activating and repressing chromatin remodeling events were apparent at different loci, but within the same effector CTL effector population, suggests that opposing regulatory mechanisms can act simultaneously at distinct gene loci.

## EPIGENETIC AND THE MAINTENANCE OF EFFECTOR FUNCTION WITHIN MEMORY T CELLS

A cardinal feature of memory T cells is their ability to elicit rapid effector function upon antigen recognition without the need for further differentiation. A number of studies have demonstrated the ability to maintain this functional capacity in the resting state is likely underpinned by maintenance of permissive epigenetic signatures at key gene loci. For example, virus-specific memory CD8^+^ T cells exhibit an open chromatin structure with enrichment of H3K4me3, H3K9ac and loss of H3K27me3, at the *ifnG*, *tnfA*, *gzmB*, and *pfp* effector gene loci (Northrop et al., 2006, 2008; Araki et al., 2008, 2009; Denton et al., 2011; Zediak et al., 2011).

**FIGURE 3:**
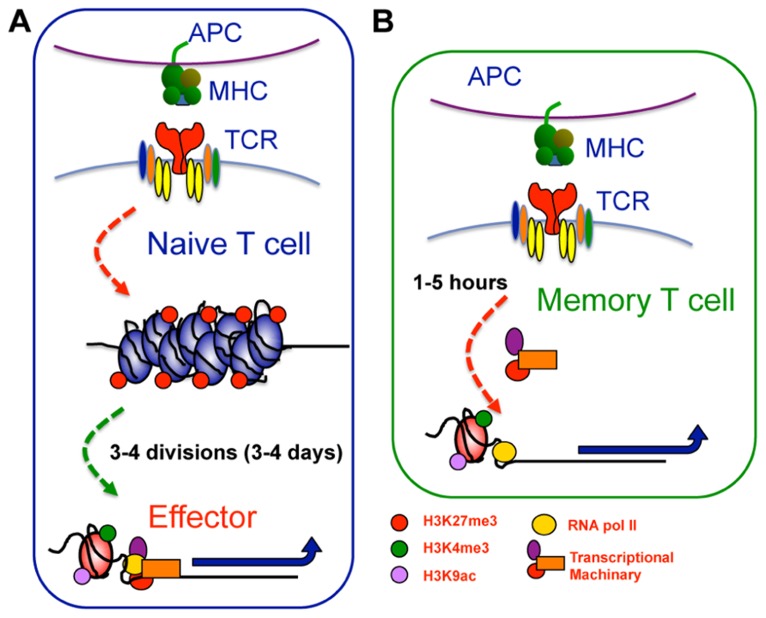
**Epigenetic reprogramming within key loci of memory T cells enables rapid effector function upon restimulation**. **(A)** Within naive CTL, key effector gene loci, such as IFN-γ or gzmB, exhibit repressive epigenetic signatures characterized by a heterochromatin structure, deposition of H3K27me3, and lack of H3K4me3 and H3K9ac. Upon T cell activation, these gene loci undergo significant chromatin remodeling becoming more accessible and acquiring a permissive epigenetic signature characterized by loss of H3K27me3, and deposition of H3K4me3 and H3K9ac. This permissive signature acts as a platform allowing recruitment of gene specific TFs and the transcriptional machinery needed to drive transcription. **(B)** In memory cells, the permissive epigenetic signature at effector gene loci is maintained in the long term. The maintenance of permissive epigenetic signatures at key effector gene loci within memory T cells serves to keep RNA pol II “on-hold” at the TSS. Subsequent to TCR ligation, the RNA polymerase is released, allowing rapid gene expression and thus, rapid memory T cell effector function.

Importantly, maintenance of a permissive epigenetic signature at the IFN-γ and TNF-α gene loci within virus-specific memory T cells coincided with docking of RNA polymerase II, a core component of the transcriptional machinery, at the proximal promoter (Denton et al., 2011; Zediak et al., 2011). This “stalling” of RNA pol II at the transcriptional start site of proximal promoters is associated with genes that are “poised” or “at the ready” for transcriptional activity (Margaritis and Holstege, 2008). Thus, epigenetic re-programing that results in maintenance of permissive epigenetic signatures at key effector gene loci enables memory T cells to keep RNA pol II “on-hold” at the TSS. Subsequent to TCR ligation, the RNA polymerase is released, allowing rapid gene expression and ensuring rapid memory T cell effector function (**Figure 3**). Interestingly, not all effector gene loci appear to be “poised” in memory CD8^+^ T cells with RNA poll II docking not observed at the *gzmB*, *pfp* (Zediak et al., 2011) or *gzmA* (L. Hatton, B. E. Russ, and S. J. Turner, unpublished data) proximal promoters, despite the presence of a permissive epigenetic signature. While gzmB and gzmA expression is up-regulated in reactivated memory CD8^+^ T cells, it is delayed when compared to cytokine production (Lawrence and Braciale, 2004; Jenkins et al., 2007, 2008; Moffat et al., 2009; Denton et al., 2011; Zediak et al., 2011). Thus, it appears that different molecular mechanisms work to impart different kinetics of cytokine versus cytolytic effector gene expression within reactivated memory T cells.

A more recent study has utilized high-throughput sequencing in combination with ChIP to map genome-wide H3K4me3 and H3K27me3 deposition within polyclonal naïve and memory human CD8^+^ T cell populations (Araki et al., 2009). In general agreement with previous studies, a direct positive or negative correlation between gene expression and respective H3K4me3 or H3K27me3 deposition was observed (Araki et al., 2009). Interestingly, this genome-wide analysis also identified other correlates of gene transcriptional activity. For example, some loci within memory T cell populations exhibited H3K4me3 deposition, but only active transcription upon anti-CD3 stimulation (termed poised loci), a pattern not dissimilar to poised effector loci identified in previous analyses of virus-specific memory CTL (Denton et al., 2011; Zediak et al., 2011). Thus, epigenetic re-programing is a likely mechanism that underpins both the acquisition of lineage-specific T cell effector function and the rapid responsiveness that exhibited by memory T cells. The Araki study also suggests that different epigenetic signatures may be key in regulating different types of transcriptional responses during T cell differentiation. This is difficult to fully ascertain due to the polyclonal nature of the T cell populations analyzed. It will be of interest to examine T cell populations that have a linked differentiation history (i.e., are known to be responding to the same differentiation signals). Moreover, the T cell subsets examined in the Araki study were static populations and thus, there is no insight into the dynamics of epigenetic re-programing upon T cell activation. For example, do such changes occur quickly upon activation and do they all require cellular division?

## SIGNALS THE DRIVE THE DYNAMIC CHANGES IN EPIGENETIC SIGNATURES WITHIN ACTIVATED T CELLS

Interestingly, there are few studies that have identified the specific signals that shape the epigenetic re-programing of T cell differentiation in response to activation. It is appreciated that the provision of both co-stimulatory and cytokine signals at the time of CTL activation promotes up-regulation of specific transcriptional programs associated with full maturation of effector and memory CD8^+^ T cell responses (Agarwal et al., 2009). In particular, the combination of IL-12 and IFN-α signaling promotes up-regulation of effector genes such as gzmB, IFN-γ, and the TFs, T-bet, and Eomes (Agarwal et al., 2009). Importantly, up-regulation of signature CD8^+^ T cell effector genes in response to these third signals is associated with chromatin remodeling and an increase in histone acetylation within these effector and TF gene loci (Agarwal et al., 2009). Thus, IL-12 and type I IFN signals induced the appropriate chromatin remodeling events required to promote increased transcriptional activity at those gene loci key for both effector and memory T cell differentiation.

As mentioned earlier, provision of CD4^+^ T cell help is essential for the establishment and maintenance of CD8^+^ T cell memory. Given the dynamic epigenetic remodeling that occurs with memory T cell differentiation, CD4 help likely plays a key role in establishing the appropriate permissive epigenetic signatures within effector gene loci of memory T cells. This is supported by the observation that memory virus-specific CD8^+^ T cells, generated in the absence of CD4^+^ T cell help, had diminished histone acetylation at the *ifnG* gene, with this correlating with decreased IFN-γ expression (Northrop et al., 2006, 2008). It will be of particular interest to determine the extent to which a lack of CD4^+^ T cell help contributes to inappropriate epigenetic re-programing during virus-specific memory T cell differentiation. Does such help only result in remodeling of a limited number of gene loci, or are there broader consequences? We are currently utilizing ChIP-seq approaches to examine genome-wide changes in the epigenome between “helped” and “unhelped” memory T cells to help pinpoint both the precise mechanisms, and gene loci within virus-specific T cells that undergo CD4-dependent epigenetic re-programing upon activation.

Questions remain regarding what the precise enzymes and transcription factors are that come to together to rewrite the epigenetic signature during T cell differentiation. The answers to these questions will be important if such processes are ever to be targeted for the optimization (in the case of vaccine strategies for example) or the attenuation (in the case of autoimmune disease) of T cell immunity. One thing that is clear is that with the recent advances in systems biology approaches, our appreciation of just these molecular mechanisms fine tune our T cell immune responses can only grow and will provide new opportunities to think about how to best harness T cell immunity to fight infection, treat cancer and ameliorate autoimmune disease.

## Conflict of Interest Statement

The authors declare that the research was conducted in the absence of any commercial or financial relationships that could be construed as a potential conflict of interest.
